# Frailty prevalence and association with mortality across birth cohorts in Swedish registry data

**DOI:** 10.1093/geroni/igab046.3000

**Published:** 2021-12-17

**Authors:** Alexandra Wennberg, Karin Modig

**Affiliations:** Karolinska Institutet, Stockholm, Stockholms Lan, Sweden

## Abstract

Frailty is associated with poor health outcomes, reduced quality of life, and mortality. To understand how prevalence of frailty may have changed across birth cohorts, we investigated frailty prevalence at ages 75, 85, and 95 in people born in 1910, 1920, and 1930 in Swedish national registry data. Frailty was assessed with the Hospital Frailty Risk Score, a weighted sum of 109 ICD codes, which we calculated for each year leading up to the specified ages. We additionally investigated the association between frailty and mortality in these birth cohorts. We observed, at 75, a decrease in prevalence of frailty across birth cohorts (16.9%, 10.8%, and 8.8%, respectively). Interestingly, at 85, we found a U-shaped pattern, where those born in 1920 (14.1%) had lower prevalence of frailty than those born in either 1910 (27.7%) or 1930 (25.1%). At age 95, we saw a low prevalence of frailty in the 1910 (7.3%) and 1920 (3.8%) birth cohorts –potentially because of selective survival. There were not substantial differences in prevalence of frailty by sex or birth country. In Cox proportional hazard models adjusted for sex, frailty was consistently associated with mortality. We observed the greatest hazard ratios in the 1930 birth cohort at 75 (HR=2.79, 95% CI 2.62, 2.97) and 85 (HR=2.26, 95% CI 2.01, 2.53) and the 1920 birth cohort at 75 (HR=2.19, 95% CI 2.09, 2.29), where risk was double that of those who were not frail. Understanding changes in prevalence of frailty will help inform public health and intervention measures.

